# Study on acupuncture improving sleep deprivation comorbid with cognitive dysfunction based on rs-fMRI: A protocol for systematic review and meta-analysis

**DOI:** 10.1097/MD.0000000000033490

**Published:** 2022-04-07

**Authors:** Xiaole Guo, Weiwan Yang, Ying Wang, Shiqi Ma, Qi Lu, Hongfeng Wang

**Affiliations:** a Department of Acupuncture and Tui Na, Changchun University of Chinese Medicine, Changchun, China; b Changchun University of Chinese Medicine, Changchun, China.

**Keywords:** acupuncture, cognitive dysfunction, meta-analysis, protocol, rs-fMRI, systematic review

## Abstract

Sleep deprivation often lead to changes in attention, memory, mood, alertness, and metabolism. Especially, it is often accompanied by cognitive impairment of the brain. Acupuncture is safe and effective for improving cognitive function, but its underlying mechanism is not fully understood. Resting-state functional magnetic resonance imaging is an important means to study brain activity changes. However, the results are inconsistent and lack systematic evaluation and analysis.

**Methods:**

We will search 9 databases, including PubMed, EMBASE, EBSCOhost-Medline, Web of Science, Cochrane Library, China National Knowledge Infrastructure, VIP Database and Wan-Fang Database, Chinese Biomedical Literature Database, and 2 clinical trials register platforms: Chinese Clinical Trial Registry, ClinicalTrials.gov (www.ClinicalTrials.gov/) from inception to November 1, 2022. We will use the Review Manager 5.4 software provided by the Cochrane Collaborative Network for statistical analysis. We then assessed the quality and risk of the included studies and observed the outcome measures.

**Results:**

This study will analyze the effect of acupuncture on brain activity changes, improvement of sleep duration, and cognitive impairment.

**Conclusion:**

This meta-analysis aims to investigate the efficacy of acupuncture on brain activity changes in sleep deprivation comorbid with cognitive dysfunction, so as to provide effective evidence for clarifying its pathogenesis.

## 1. Introduction

Sleep is an important physiological phenomenon of the human body, and normal sleep is an important guarantee for maintaining a healthy state of life and basic life activities. Sleep quality directly affects people's behavior, cognition, and even physical and mental health. With the acceleration of the pace of life and the increasing pressure from external factors such as society, environment, and working style, sleep deprivation (SD) has become the new normal of modern people, and the overall sleep duration has been on a long-term downward trend globally.^[1]^ According to the sleep status survey of adult residents in 15 provinces of China in 2015, 9.8% of adults have the problem of sleep deficiency,^[2]^, especially for a series of workers with occupational characteristics such as high load, shift work, and sleep disorders, the sleep ratio is seriously disordered.^[3]^ Previous studies have shown that SD can cause individual abnormalities in emotion, learning, and memory,^[4–6]^ such as mood change, memory decline, alertness, attention decline, executive decision-making ability decline, and so on, leading to accidents and diseases.^[7]^ Therefore, how to reduce the negative impact of SD on the cognitive dysfunction of workers in all walks of life has become a key issue to be solved.

The pathogenesis of SD associated with cognitive disorders is not very clear at present, in modern medicine, awakening promoters, and receptor inhibitors are the main drugs to promote sleep and improve cognition.^[8,9]^ Although these drugs can extend the patients’ sleep time and improve cognition, they cannot solve the underlying problem of sleep deficiency and have great side effects in the long term. As the treasure of China, traditional Chinese acupuncture has a long history, safety, and effectiveness, and has been widely respected in clinical application. A large number of animal experiments and related clinical studies have confirmed the safety and effect of acupuncture in the treatment of SD with cognitive dysfunction,^[10,11]^ but the specific mechanism remains to be further explored.

Resting-state functional magnetic resonance imaging (rs-fMRI) is a commonly used technique to study brain function. rs-fMRI uses magnetic resonance equipment to detect changes in blood oxygen levels during brain activity, so as to identify and locate brain functional areas and provide strong evidence for the study of complex brain activities.^[12]^ It is one of the most commonly used technical means for brain function research at present. Studies using rs-fMRI have shown that SD is associated with arousal, emotion, reward, and abnormal functional activity in cognitive areas of the brain.^[13]^ It is not only associated with abnormal functioning of local brain regions but also with abnormal internal connections of the default network, the prominence network, and the emotional network.^[14]^ rs-fMRI has been used to study the mechanism of acupuncture in treating diseases.^[15]^ Due to the different testing standards and sample sizes of testing equipment and analysis software, the results are inconsistent. Therefore, the meta-analysis method was used in this study to conduct an integrated analysis of the changes of low-frequency fluctuation amplitude (ALFF) and regional homogeneity (Re Ho) and to explore the characteristics and rules of the changes of brain activity in patients with cognitive dysfunction induced by SD with acupuncture, so as to provide effective evidence for clarifying the pathogenesis.

## 2. Methods and analysis

The protocol of this meta-analysis will be conducted and reported in accordance with the Preferred Reporting Items for Systematic Reviews and Meta-Analysis Protocols statement guidelines.^[16]^ This systematic review protocol was registered in PROSPERO (CRD 42022375662).

### 2.1. Study inclusion criteria

#### 2.1.1. Types of studies.

This review will include randomized controlled trials reporting that study on acupuncture improving cognitive dysfunction in patients with SD based on rs-fMRI. Randomized controlled trials comparing pharmacotherapy, sham acupuncture, or placebo will be included. All eligible trials will be included regardless of language and publication type. Articles of the following research types will be excluded: case series, observational studies (including cohort and case-control studies), and retrospective studies, qualitative studies, animal experiments, review articles. There are no restrictions on the study area, race, patient age, and gender.

#### 2.1.2. Participants.

Participants meeting the SD criteria will be included, including acute, chronic SD, and complete or partial SD, regardless of age, gender, and case source. Patients need to have a certain degree of cognitive dysfunction, such as decreased learning and memory ability, alertness, attention, and execution (secondary to SD).

#### 2.1.3. Interventions.

Intervention methods include body acupuncture (hand/electricity), ear acupuncture, scalp acupuncture, and press acupuncture, regardless of acupuncture techniques and simulation methods. The combination of non-stimulating acupoints and Western medicine treatment is excluded.

#### 2.1.4. Comparisons.

The control group will receive any kind of treatment without acupuncture and moxibustion (Western medicine, placebo, or regular treatment).

#### 2.1.5. Outcomes.

The results include efficacy and safety evaluation. The primary outcome measures included Re Ho and ALFF of the whole brain, which were evaluated by fMRI; Pittsburgh Sleep Quality Index and Montreal cognitive assessment scale were used to assess the changes in sleep quality and cognitive function; vigilant attention was measured using a psychomotor vigilance test. The secondary outcome measures included Hamilton Depression Scale, Hamilton Anxiety Scale, and adverse reactions.

### 2.2. Search methods

We will search 9 databases, including PubMed, EMBASE, EBSCOhost-Medline, Web of Science, Cochrane Library, China National Knowledge Infrastructure, VIP Database and Wan-Fang Database, Chinese Biomedical Literature Database, and 2 clinical trials register platforms: Chinese Clinical Trial Registry, ClinicalTrials.gov (www.ClinicalTrials.gov/) from inception to November 1, 2022. We will use the Review Manager 5.4 software provided by the Cochrane Collaborative Network (Oxford, England) for statistical analysis. We then assessed the quality and risk of the included studies and observed the outcome measures. The search term will consist of 4 parts: study type, disease, intervention method, and technical means: (“randomized controlled trial” or “randomized ” or “randomly” or “trial” or “clinical trials”) and (“sleep insufficient” or “sleep deprivation” or “sleep disorders” or “sleep loss” or “insomnia” or “dyssomnias”) and (“cognitive dysfunction” or “cognitive impairment” or “cognitive decline”) and (“acupuncture therapy” or “acupuncture” or “acupoints” or “body acupuncture” or “electroacupuncture” or “scalp acupuncture” or “manual acupuncture” or “auricular acupuncture” or “ear acupuncture” or “press acupuncture”) and (“magnetic Resonance Imaging” or “functional magnetic resonance” or “resting state functional magnetic resonance imaging” or “resting-state fMRI”). Chinese translations of these search terms will be used for the Chinese databases. The search strategy for the PubMed database is shown in Table 1. The search strategy for other online databases will be adjusted according to their requirements.

**Table 1:** Search strategy for PubMed.

Number	Search terms
#1	Randomized controlled trial. [pt]
#2	Randomised. [ti,ab]
#3	Placebo. [ti,ab]
#4	Randomly. [ti,ab]
#5	Sham. [ti,ab]
#6	Trial. [ti,ab]
#7	Clinical trials as topic. [MeSH]
#8	t#1 OR #2 OR #3 OR #4 OR #5 OR #6 OR #7
#9	Human. [MeSH]
#10	#8 AND #9
#11	Sleep deprivation. [MeSH]
#12	Sleep insufficient. [MeSH]
#13	Sleep loss. [MeSH]
#14	Sleep disorders. [ti,ab]
#15	Dyssomnias. [ti,ab]
#16	Insomnia. [ti,ab]
#17	#11 OR #12 OR #13 OR #14 OR #15 OR #16
#18	Cognitive dysfunction
#19	Cognitive impairment
#20	cognitive decline
#21	#18 OR #19 OR #20
#22	#17 AND #21
#23	Acupuncture therapy. [MeSH]
#24	Acupuncture. [ti,ab]
#25	Acupoints. [ti,ab]
#26	Body acupuncture. [ti,ab]
#27	Scalp acupuncture. [ti,ab]
#28	electroacupuncture. [ti,ab]
#29	manual acupuncture. [ti,ab]
#30	auricular acupuncture. [ti,ab]
#31	ear acupuncture. [ti,ab]
#32	press acupuncture. [ti,ab]
#33	#23 OR #24 OR #25 OR #26 OR #27 OR #28 OR #29 OR #30 OR #31 OR
#34	#32
#35	Magnetic Resonance Imaging. [MeSH]
#36	Functional magnetic resonance. [ti,ab]
#37	Resting state functional magnetic resonance imaging. [ti,ab]
#38	Resting-state fMRI. [ti,ab]
#39	MRI. [ti,ab] OR fMRI. [ti,ab] OR BOLD-MRI. [ti,ab] OR rs-fMRI. [ti,ab] #34 OR #35 OR #36 OR #37 OR#38
#40	#10 AND #22 AND #33 AND #39

### 2.3. Data collection and management

#### 2.3.1. Selections of studies.

**Figure 1.**
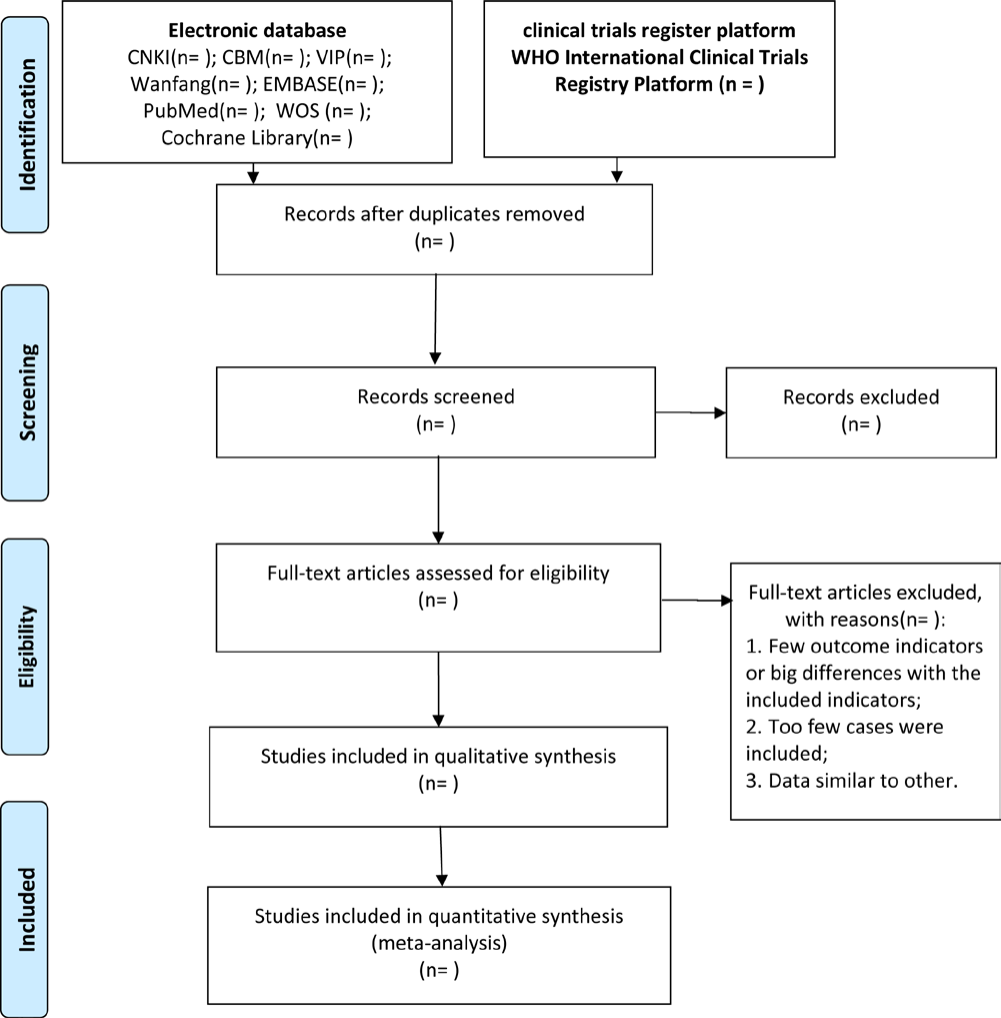
PRISMA flow diagram of study and exclusion. PRISMA, Preferred Reporting Items for Systematic Reviews and Meta-Analyses.

Clinical studies will be identified and reviewed by 2 independent reviewers (QL and YW). In order to ensure that the reviewers have a good understanding of the background and purpose of the review, they will be trained in advance. Use Note Express 3.2.0 software (http://www.inoteexpress.com/aegean/) to independently manage the search results from the above-mentioned databases. First, remove the duplicate literature. Afterward, after reviewing the title and abstract, exclude the literature that does not comply with the standard. Finally, further review the full text and rule out the literature that is not in line with the standard. Any disagreements related to study selection results will be discussed after cross-checking and arbitrated by the third reviewer (W.Y.). The flow diagram of all study selection procedures is shown in Figure 1.

#### 2.3.2. Data extraction.

Two reviewers (QL and YW) will independently select literature and extract data in accordance with the retrieval strategy. If there are differences of opinion, the third reviewer (X.G.) will participate in the discussion. According to the designed data extraction table, the basic information of the study includes:

1. Identification information (article title, publication time, first author, and journal);
2. General information (country, type of study, sample size, test design, and diagnosis standard);
3. Participants (age, sex, and pretreatment fMRI results);
4. Intervention measures (acupuncture type, point selection, and treatment frequency/course of treatment/duration; administration time);
5. Comparator (if any, details of treatment, including treatment method, frequency, and course of treatment);
6. Results (time, scale, and fMRI result points of each measurement, including Re Ho and ALFF, Pittsburgh Sleep Quality Index, Montreal cognitive assessment, psychomotor vigilance test, Hamilton Depression Scale, Hamilton Anxiety Scale, follow-up time, and adverse events).

### 2.4. Risk of bias assessment

Two independent reviewers (S.M. and W.Y.) will assess the risk of bias with the Cochrane Risk of Bias Tool according to the Cochrane Handbook 5.1.0 for Systematic Reviews of Interventions. The 2 reviewers will assess 7 items, which consist of the risk of bias of sequence generation, allocation concealment, blinding of participants personnel and outcome assessment, incomplete outcome data, selective outcome reporting, and other biases. If there is disagreement during the assessing process, 2 reviewers will discuss or consult the third reviewer (X.G.) for a decision. Three evaluation grades are low, unclear, and high risk of bias.

### 2.5. Measures of treatment effect

Mean differences with 95% confidence intervals will present as continuous data. Also, the risk ratio will be the expression of dichotomous data.

### 2.6. Dealing with missing data

We will e-mail the corresponding author to obtain the necessary information, which is missing or insufficient. Otherwise, we will analyze the existing information and conduct a sensitivity analysis to address the potential impact of missing data.

### 2.7. Assessment of heterogeneity

*I*^2^ will be used for assessing statistical heterogeneity. It is acknowledged that *I*^2^ < 25% indicates negligible heterogeneity, 25% < *I*^2^ < 50% indicates mild heterogeneity, 50% < *I*^2^ < 75% moderate heterogeneity, and *I*^2^ ≥ 75% high heterogeneity.

### 2.8. Assessment of reporting bias

Over 10 studies included, we will take advantage of funnel plot to assess the reporting bias. Symmetrical funnel indicates no publishing bias, but if the funnel is not symmetrical, which indicates publishing bias exists. *P* value will be utilized, while <10 studies included.

### 2.9. Data syntheses

We will take advantage of Rev Man software (version 5.4) for Statistical analyses performing. Only if there is no or mild significant heterogeneity (*I*^2^), we will apply the fixed-effect model, or the random-effects model will be selected.

### 2.10. Analysis of subgroups or subsets

Subgroup analysis will be conducted to evaluate the specific influence of intervention type, acupoints selection, and treatment duration on pooled results. If the data is insufficient, qualitative synthesis will be conducted instead of quantitative synthesis.

### 2.11. Sensitivity analysis

The robustness of the results will be assessed by sensitivity analysis performance which will focus on the processing method of missing data.

### 2.12. Grading the quality of evidence

According to the Grading of Recommendations Assessment, Development, and Evaluation method, 2 reviewers (W.Y. and X.G.) will use the Grading of Recommendations Assessment, Development, and Evaluation rating standards to independently assess the quality of evidence for each outcome as 4 levels: “very low,” “low,” “moderate,” or “high” quality. The quality of evidence will be assessed according to the risk of bias, inconsistency, indirectness, imprecision, and publication bias.

### 2.13. Ethics and dissemination

Due to nothing of the information will be obtained from an individual participant, the systematic review does not need ethical approval or patient consent. The results of this meta-analysis will be published in peer-reviewed scientific journals.

## 3. Discussion

Currently, it is believed that the neurobiological mechanism of post-SD cognitive impairment is mainly through changing the metabolism of brain substances to structural and functional damage neurons in different brain regions, and thus impair cognitive function.^[17]^ Studies on the mechanism of SD influence on cognitive function based on fMRI technology mainly focus on the brain functional connection and its connection with cognitive function and other neurobehavioral studies. For example, in the study on the effect of SD on alertness and attention function based on fMRI technology, it was found that SD had a negative effect on the attention function network, and the mechanism might be that SD weakened the functional connection between the dorsal attention network system of the brain and other brain regions.^[18,19]^ In terms of learning and memory, someone^[20]^ studied short-term memory disorders with the hippocampus as the seed point and found that SD reduced the functional connections between the hippocampus and superior frontal gyrus, temporal lobe, and auxiliary motor area, indicating that the abnormal functional connections between hippocampus and cortex after SD were related to the reduction of short-term memory. In addition, studies have shown that^[21]^ SD could lead to the dysfunction of the functional connectivity of the hippocampus, thus affecting the process of memory consolidation and damaging long-term memory. In terms of executive decision-making ability, it is reported^[22]^ that the accuracy of recognizing neutral and positive words in the SD population decreased, and the activation of the dorsolateral frontal cortex was enhanced when recognizing positive words, while the activation of the insula was enhanced when recognizing emotional words, indicating that SD can affect the judgment, decision making, and executive ability, and is closely related to the dorsolateral frontal cortex and insula in the brain. In conclusion, rs-fMRI is an important brain imaging technique for the study of cognitive dysfunction in SD patients.

The application of acupuncture can improve sleep quality, reduce the occurrence of adverse events related to sleep disorders, and alleviate the negative effects of SD on cognitive function, which is safe and effective. Previous studies have reported that acupuncture San Yin Jiao Point (SP6)^[23,24]^ can improve the activity intensity of the default network, precuneus anterior lobe, cingulate gyrus, and other brain regions, and strengthen the interaction between the frontal lobe and temporal lobe, and the connection of synchronous neural network, indicating that acupuncture of SP6 has a strong ability of integration and regulation of mental intelligence, emotion regulation, and brain cognitive dysfunction.

In recent years, relevant researches on exploring the mechanism of acupuncture for SD comorbid with cognitive decline on rs-fMRI have been reported, but there has been no systematic review and meta-analysis. This study will integrate all the latest relevant literature in this field to explore the effects of acupuncture on the brain activity of patients with SD and cognitive dysfunction. However, due to the lack of relevant clinical research literature, correlation analysis such as subgroup analysis and sensitivity analysis may not be possible. In addition, heterogeneity may exist between studies due to the use of different evaluation criteria and intervention methods. In conclusion, the purpose of this systematic review and meta-analysis is to provide an evidence-based medical basis for clarifying the central nervous system mechanism of acupuncture in the treatment of SD comorbid with cognitive dysfunction.

## Acknowledgment

We appreciate the financial support received from the Natural Science Foundation of Jilin Province (No. YDZJ202101ZYTS103)

## Author contributions

**Conceptualization:** Xiaole Guo.

**Data curation:** Ying Wang, Qi Lu.

**Formal analysis:** Weiwan Yang, Shiqi Ma.

**Funding acquisition:** Hongfeng Wang.

**Investigation:** Xiaole Guo, Weiwan Yang.

**Supervision:** Hongfeng Wang.

**Validation:** Ying Wang, Shiqi Ma.

**Writing – original draft:** Xiaole Guo.

**Writing – review & editing:** Xiaole Guo, Hongfeng Wang.
